# Is universal depression screening cost-effective across all Indian states? Addressing parameter uncertainty

**DOI:** 10.1016/j.lansea.2025.100712

**Published:** 2026-01-05

**Authors:** Ziyi Niu, Wenyan Tang, Jiafeng Zhang

**Affiliations:** a Department of Endocrinology, No. 905 Hospital of PLA Navy, Naval Medical University, Shanghai, 200052, China; b College of Basic Medical Sciences, Naval Medical University, Shanghai, 200433, China

We read with great interest the article by Purohit et al. evaluating the cost-effectiveness of universal depression screening in India's primary healthcare system.^1^ The study addresses a critical evidence gap for mental health policy in low- and middle-income countries. However, there are some limitations to cons.

**First, the representativeness of utility values merits attention.** The EQ-5D-5L utility scores were derived from 259 patients attending the psychiatry outpatient department of a tertiary care hospital. This sampling approach risks selection bias, as patients attending a tertiary hospital psychiatry likely represent more severe cases or individuals with greater treatment-seeking propensity compared to those identified through community-based screening.^2^ Patients detected via population screening often include asymptomatic or mildly symptomatic individuals who may report higher baseline utility values. Consequently, the incremental utility gain attributable to screening and treatment could be overestimated. Given that quality-adjusted life years (QALYs) constitute the primary outcome measure in cost-utility analysis, even modest deviations in utility parameters could substantially alter the incremental cost-utility ratios (ICURs). We suggest that future validation studies measure utilities directly in community-screened populations, or that broader sensitivity analyses be conducted around these parameters.

**Second, the treatment adherence assumptions appear optimistic.** The model incorporates a 10% loss-to-follow-up rate during the treatment phase, implying approximately 90% treatment retention rate. However, published evidence consistently demonstrates that real-world adherence to antidepressant therapy is considerably lower. A systematic review found that six-month non-adherence rates averaged 46–52% across psychiatric and primary care populations.^3^ More recent real-world data from population-based cohorts report overall adherence rates as low as 31% over 12 months.^3^ These challenges may be more pronounced in resource-limited settings where transportation difficulties, medication-related stigma, and limited follow-up infrastructure are prevalent. The treatment efficacy parameters employed in the model were derived from clinical trials, which generally achieve higher adherence rates than real-world practice. If treatment adherence were adjusted to reflect empirical estimates from similar settings, the effective treatment benefit—and consequently the QALY gains—would be proportionally reduced. This could shift the ICUR toward or beyond the cost-effectiveness threshold. We recommend that the authors conduct threshold analyses to identify the minimum adherence rate at which the intervention remains cost-effective.

**Third, the substantial heterogeneity across Indian states warrants consideration.** The analysis employs nationally aggregated parameters to generate unified cost-effectiveness conclusions. However, India's states exhibit marked variation in mental disorder prevalence. Data from the Global Burden of Disease Study 1990–2017 reveal that the age-standardised prevalence of depressive disorders—the intervention's target—varied by more than threefold across states, from 1.46% in Assam to 4.63% in Himachal Pradesh. Similarly, the prevalence of any mental disorder showed a 2.4-fold difference, ranging from 6.64% per 100,000 in Nagaland to 16.02% in Manipur.^4^ Beyond prevalence, states differ substantially in primary healthcare infrastructure density, mental health workforce availability, healthcare-seeking behaviours, and economic development levels. A screening strategy deemed cost-effective at the national level may not be uniformly efficient across all states. In states with lower disease burden or inadequate treatment capacity, the intervention could yield diminished returns or even prove cost-ineffective. Conversely, states with high prevalence but strong primary care networks may derive greater benefit. State-level stratified analyses or, at a minimum, subgroup analyses by regional characteristics would enhance the policy relevance of these findings and support more targeted resource allocation decisions.

In conclusion, while Purohit et al. provide valuable evidence supporting depression screening integration into India's primary healthcare system, the aforementioned methodological considerations suggest that the reported cost-effectiveness estimates should be interpreted with appropriate caution. Addressing these limitations through additional sensitivity analyses, empirical validation studies, and disaggregated regional assessments would strengthen the evidence base for policy implementation. We believe these refinements would further enhance the considerable contribution this study makes to global mental health economics.

## Contributors

Ziyi Niu: writing–original draft, data collection, data analysis, data interpretation; Wenyan Tang: writing–original draft, data collection, data analysis, data interpretation; Jiafeng Zhang: writing–review & editing, resources, supervision.

## Declaration of interests

The authors declare that they have no competing interests.
